# Exploring the linguistic complexity of third-grade numerical literacy

**DOI:** 10.1186/s41235-024-00575-5

**Published:** 2024-07-18

**Authors:** Ella Shalit, Dror Dotan

**Affiliations:** Mathematical Thinking Lab, School of Education and the Sagol School of Neuroscience, Tel Aviv University, 6997801 Tel Aviv, Israel

**Keywords:** Number syntax, Number reading, Development of numerical skills, Conceptual versus procedural knowledge

## Abstract

Reading numbers aloud, a central aspect of numerical literacy, is a challenging skill to acquire, but the origins of this difficulty remain poorly understood. To investigate this matter, we examined the performance of 127 third- and fourth-grade children who read aloud, in Hebrew, numbers with 2–5 digits. We found several key observations. First, we observed a substantial variation among the 3rd graders—7% and 59% errors in the top and bottom deciles, respectively. Second, the task difficulty stemmed from syntactic processing: Most errors were distortions of the number’s syntax, as opposed to digit substitutions or transpositions, and the main factor affecting a specific number’s difficulty was not its magnitude, as is commonly assumed, but rather its syntactic structure. Third, number reading performance was not predicted by a school-like task that assessed syntactic-conceptual knowledge of the decimal system structure, but rather by knowledge of specific syntactic-verbal rules, suggesting that the syntactic-verbal knowledge is separate from the syntactic-conceptual knowledge. Last, there was a double dissociation between 4-digit numbers and 5-digit numbers, which in Hebrew have completely different syntactic structures: Half of the children showed a significant advantage in one number length compared to the other, with equal numbers of children preferring either length. This indicates that the different syntactic-verbal rules are learned relatively independently of each other, with little or no generalization from one rule to another. In light of these findings, we propose that schools should specifically teach number reading, with focus on specific syntactic-verbal rules.

**Supplementary Information:**

The online version contains supplementary material available at 10.1186/s41235-024-00575-5.

## Introduction

The ability to handle symbolic numbers (digits and number words), namely to read, write, say, and understand multi-digit numbers, is a fundamental skill in the mathematical domain. Handling numbers as symbols is separate from the ability to understand their meaning as representing magnitudes (Dehaene et al., 2003; Yuan et al., 2019), and it is a major predictor of elementary school arithmetic abilities (Banfi et al., 2022; Guerrero et al., 2020; Habermann et al., 2020; Moeller et al., 2011). However, learning to handle multi-digit symbolic numbers is not only important, but it is also hard. In the first school grades, children make many errors when they read and write multi-digit numbers (Steiner et al., 2021), and it takes a long time until they master the decimal number system. For example, although they begin to understand the place-value principle, which dictates that the same digit has different meanings in different decimal positions, at a relatively young age (Mix et al., 2014), it can still take years until they reach a full understanding of how the place-value principle should be used to interpret the meaning of multi-digit numbers or in simple arithmetic calculations (Cheung & Ansari, 2021; Fuson, 1990; Fuson & Briars, 1990). It may take even longer until children can process automatically and efficiently the relative quantities represented by digits in different positions (Dotan & Dehaene, 2016).

The present study examined number reading in children who are still in the process of learning this skill. In everyday speech, the term “number reading” is used to describe different tasks—e.g., reading aloud, comprehension, magnitude evaluation, and more. Here, we focused on the processing of numbers as symbols (as opposed to magnitudes), and we examined the specific case of reading aloud multi-digit numbers, which is a major predictor of arithmetic skills (Habermann et al., 2020).

### Why is it hard to learn to read numbers? The effects of syntax and magnitude

A major source of difficulty in reading multi-digit numbers lies in the need to handle the numbers’ syntactic structure—the various inter-relations between the digits or number words in the number. When treating numbers as magnitudes, these relations are reflected in the place-value principle, but for symbolic-only processing, they consist of information such as how many digits a digit string has, and of syntactic irregularities such that 0 is not manifested verbally. When reading or writing numbers, these syntactic aspects pose a considerable challenge for children (Barrouillet et al., 2004; Batista et al., 2023; Fuson, 1990; Moura et al., 2013; Power & Dal Martello, 1990, 1997; Seron & Fayol, 1994; Steiner et al., 2021; Zuber et al., 2009) and even for adults (Handelsman & Dotan, 2023).

Several types of evidence indicate this syntactic challenge. First, when analyzing the types of errors in reading, a common finding is that there are fewer lexical errors, i.e., distortions of a specific digit’s identity (e.g., reading 234 as 239), and more syntactic errors, i.e., distortions of the number’s syntactic structure, e.g., reading 234 as 2034 (Handelsman & Dotan, 2023; Moura et al., 2013; Steiner et al., 2021). Second, when analyzing specific items, some studies reported more errors in numbers whose syntactic structure is complex—e.g., numbers with the digit 0, which creates a syntactic irregularity because this digit is not verbalized (Delazer & Denes, 1998; Furumoto, 2006; Granà et al., 2003). Third, when examining individuals with impaired number reading (dysnumeria), there are more cases of dysnumeria whose origin is a deficit in a syntactic cognitive process than cases of deficits in non-syntactic processes (Handelsman & Dotan, 2023).

Another origin of difficulty in reading numbers, which was probably discussed in the literature even more than syntax, is the number’s magnitude: Larger numbers are harder. In calculation, this phenomenon is well documented in the so-called problem size effect. Since this effect was highlighted over 50 years ago (Groen & Parkman, 1972), numerical magnitude was repeatedly shown to be a central predictor of performance in arithmetic tasks (Zbrodoff & Logan, 2005). In magnitude-processing tasks, this issue was examined even more extensively, and the common explanation is that the cognitive representation of larger magnitudes is fuzzier, which makes them less distinguishable (Dehaene, 1997). Importantly, the number’s magnitude affects the performance not only in calculation and magnitude-processing tasks but also when reading numbers aloud (Brysbaert, 1995). This effect is not trivial because reading numbers aloud requires only the two symbolic representations of numbers, namely digits and words (Cohen & Dehaene, 1991, 2000). However, it is possible that the number’s magnitude representation, or another semantic representation that correlates with magnitude, is activated automatically in some cases, either to mediate the conversion between digits and words (Cohen et al., 1994; Fias, 2001) or as an additional, non-mediating representation.

Which of these two—syntax and magnitude—is the central origin of difficulty when children learn to read numbers (or perhaps the two are equally challenging)? The existing literature does not answer this question, for at least two reasons. First, most studies (including from our lab) examined either the effect of syntax or that of magnitude, but they did not compare directly the two effects one against the other. Second, contrasting syntax against magnitude is hard because, in Latin languages, in which most research on numerical cognition was done, syntax and magnitude are confounded: Numbers with more digits are numerically larger but they are also considered more complex syntactically.^1^ In contrast, in Hebrew, in which the present study was done, syntactic complexity and magnitude are not as correlated, because the Hebrew number system has several syntactic irregularities in smaller numbers. For example, the syntactic structure of 4-digit numbers is different from that of 5- and 6-digit numbers and could perhaps be harder (below, we explain in more detail the precise syntactic structures). Moreover, even for a single number length, 4-digit numbers, the numbers 2,000–2999 have an irregular and potentially more complex syntactic-verbal structure, which is different from that of the larger 4-digit. Similarly, in 5-digit numbers, the numbers 10,000–10,999, again the smallest ones, have an irregular syntactic-verbal structure. We capitalized on these properties of Hebrew to arbitrate between the effects of syntax and magnitude.

Comparing the effects of syntax and magnitude is an important cognitive question but it could also have important pedagogical implications because, in several countries, the education system seems to have already taken a position on the syntax/magnitude question in favor of the latter. In Israel, in which the present study was run, the math curriculum defines the progression of learning the base-10 number system, and this definition completely conforms to magnitude—i.e., smaller numbers first, larger numbers later (Israel Ministry of Education, 2006). The situation is similar in the UK (UK Department for Education, 2021) and in several other countries. While it is certainly possible that this magnitude-driven approach was not motivated by educators’ specific views about how children should learn to read numbers, but by their views about how children should learn to calculate, presumably this curriculum also affects the progression of learning to read and write numbers.

### What does it take to learn to read numbers?

Another way to examine the development of number reading is by asking which kind of learning is required to develop this skill, or which kind of knowledge constitutes proficient number reading (the two questions are not identical, but they do overlap). Questions similar to this were answered by the scientific literature and pedagogical practice, either explicitly or implicitly, in at least 3 different ways, which are not mutually exclusive.

The first answer, which we may call pure cognitive, highlights that proficient number reading is handled by a set of dedicated cognitive processes. Several studies examined these processes in adults, often focusing on syntactic processing (Cipolotti, 1995; Cipolotti et al., 1994; Cohen & Dehaene, 1991; Cuetos & Miera, 1998; Deloche & Willmes, 2000; Dotan & Friedmann, 2015; Furumoto, 2006; McCloskey et al., 1986; Noël & Seron, 1993). This body of research gave rise to detailed cognitive models of number reading in adults (Cohen & Dehaene, 1991; Dotan & Friedmann, 2018; McCloskey, 1992), which also describe the several processes that handle different aspects of number syntax (see review in Dotan & Brutmann, 2022). The processes described by these models are dedicated to number reading—they are separate from the processes involved in reading words (see review in Dotan & Friedmann, 2019). How these cognitive processes develop remains an open question, however, even in the absence of a concrete answer, a minimalist cognitive view could still argue that learning to read numbers merely requires developing these cognitive processes and training them to a sufficient degree of automaticity and fluency, and that such training does not depend on "higher" forms of knowledge, e.g., being able to explain how a number is verbalized or how the decimal number system is structured. In fact, according to a minimalist cognitive view, learning to read numbers does not even necessarily depend on learning to write them (e.g., in a number dictation task), because number reading and writing involve separate cognitive processes (Cipolotti et al., 1994; Cohen & Dotan, 2023; Lochy et al., 2003; McCloskey et al., 1985). However, note that these reading-writing dissociations were observed in adults, so the conclusion is not necessarily applicable to children’s learning stages.

The second answer emphasizes procedural knowledge, i.e., knowing explicitly the set of rules that dictate how a digit string should be converted into a verbal number. For example, knowing that the digit 0 does not translate into any word, and several other rules that you must know to be able to read numbers (Power & Dal Martello, 1997). While such knowledge was not often mentioned for number reading, with respect to calculation it was discussed quite often (procedural knowledge, Chiarelli et al., 2011; Girelli & Delazer, 1996; Rosca, 2009), and it is used regularly in classrooms. Below, we use the term *syntactic-verbal knowledge* to indicate such knowledge of the syntactic rules that dictate how a digit string is verbalized.

The third answer emphasizes understanding the base-10 number system. For example, understanding that the order of digits in a number is not arbitrary, but rather each digit has a decimal position, specified relative to the number’s right end, and that the same digit could be translated into different words if it appears in different decimal positions (e.g., 2 in 230 vs. 402). Several studies raised this idea of conceptual knowledge (Dehaene & Cohen, 1997; Geary, 2004; Ohlsson & Rees, 1991; Semenza et al., 1997); and common school exercises, e.g., “What is the hundreds digit in 8724?”, reflect the assumption that such knowledge matters. Below, we use the term *syntactic-conceptual knowledge* to indicate such knowledge: the specific conceptual knowledge that concerns the structure of the decimal system as a symbolic system.

For arithmetic, the 3 kinds of knowledge/processing—cognitive processing, procedural knowledge, and conceptual knowledge—are presumably separate (Dehaene & Cohen, 1997; Girelli & Delazer, 1996; Semenza et al., 1997). Note that "separate" does not necessarily entail "independent," especially if we examine how children learn. Indeed, procedural and conceptual knowledge are related, e.g., when learning arithmetic procedures (Ohlsson & Rees, 1991), and a common assumption (at least by educators) seems to be that at least some of this conceptual and procedural knowledge is needed so that children can develop proficient cognitive processing.

We propose that this 3-tier distinction is useful also to describe number reading and writing, and we hypothesize that the patterns of separate-but-related skills, observed for calculation, also exist for number reading and writing. Cognitive processing of syntax is presumably separate from syntactic-verbal and syntactic-conceptual knowledge. This is a likely assumption given the specificity of cognitive processes, and given several findings of specific number reading disorders in adults following focal brain damage, often with spared knowledge (Cipolotti & Butterworth, 1995; Cohen & Dehaene, 1991; Dotan & Friedmann, 2018; McCloskey, 1992). At the same time, it seems almost trivial to assume that at least some syntactic-verbal knowledge, syntactic-conceptual knowledge, or both, can support children’s learning and allow them to develop proficient number reading. Here, we examined this issue by comparing the children’s performance in number reading against their performance in tasks that tap syntactic-verbal and syntactic-conceptual knowledge.

A separate question is how these three tiers are related to each other. We revisit this point in the Discussion.

### Individual differences in the development of number reading

Most developmental research on number reading and writing examined groups of children and reported group-level findings. Even when individual child performance was reported, the goal was often to show the progression of learning in an average case, not the variance (e.g., Power & Dal Martello, 1990, 1997). However, several important questions could be asked that concern at least two aspects of variance among children of the same age. One aspect is in terms of overall performance. This is important because we typically compare children’s performance to their peers, and it seems that many educators have an idea—reflected in the curriculum—about “what a typical third grader should be able to do.” Inter-child variance is somewhat captured by simple measures such as standard deviation, but only to a certain extent because the distribution of number reading performance is not always normal, even in adults (Handelsman & Dotan, 2023).

Another aspect of the inter-child variance is in terms of subtler patterns of number reading performance. Specifically, whether different children show different progression pathways in learning the symbolic number system. For example, do all children learn 3-digit numbers, then 4-digit numbers, and then 5-digit numbers? If numbers can be ordered according to a monotonous scale of complexity, we expect this to be the case. Such order may be determined by the number of digits, as in this example (2–3-4 digits), or by another factor (e.g., syntactic irregularities). The existence of such a monotonous complexity scale intrinsic to the numbers was not tested empirically, but it seems that at least in Israel and in the UK, the education systems have already taken a stand: The curriculum dictates that numbers should be taught from small to large, perhaps because this reflects a putative intrinsic order of complexity.

### The present study

We examined number reading by third-grade children, with few specific goals. Our first goal was to describe children’s performance and error patterns in a stage in which they are still learning to read numbers. Such descriptions were offered by a few previous studies (Ganayim et al., 2021; Moura et al., 2013; Power & Dal Martello, 1997; Steiner et al., 2021; Van Rinsveld & Schiltz, 2016). Here, we extended these studies in several ways. First, in terms of language: Except for a single study (Ganayim et al., 2021) that examined first graders’ reading of small numbers (< 100) in Arabic, children’s number reading was reported only in Latin languages. Here we examined Hebrew, a Semitic language with interesting syntactic irregularities that do not exist in common Latin languages. Second, in terms of number range: Whereas most previous studies focused on low grades (< 3) and small numbers (< 100), here we examined the children’s performance with larger numbers, up to 5 digits long. Data from adults indicate that these numbers are considerably harder, especially when their syntactic structure is irregular (Handelsman & Dotan, 2023). Third, in terms of granularity: We report the performance for each child and for each group of numbers with particular syntactic characteristics. Last, in terms of age: This is the first study that specifically focuses on 3rd graders. As we shall see, this age is informative because it captures an interesting stage in the children’s development.

Our second goal was to identify the main origin of challenge when learning to read numbers; in particular, to arbitrate between syntax and magnitude as potential origins of difficulty. We shall see that it is syntax, not magnitude, that poses the greater challenge.

Third, we asked whether all children follow the same progression pathway when they learn to read numbers—e.g., learn the 2-digit numbers, then 3 digits, then 4, etc.—or different children follow different pathways. If numbers have some intrinsic properties that make some numbers harder than others, and this property allows ordering all numbers according to a monotonously increasing degree of difficulty, we should expect all children’s learning progression to follow this order. As we shall see, this was not the case.

We focused on comparing 4-digit and 5-digit numbers because in Hebrew these numbers have different syntactic structures, which could potentially be relatively independent of each other. The focus on these numbers was one of the reasons for which we examined 3rd-grade participants: Our pilot studies suggested that at this grade, children have intermediate knowledge of 4- and 5-digit numbers, i.e., they already know in principle how these numbers should be read, but their performance is still sufficiently far from ceiling. Moreover, the math curriculum (Israel Ministry of Education, 2006) dictates that by the end of grade 3, children should know how to handle 4-digit numbers but not yet 5-digit numbers, so focusing on the 3rd grade allows for an interesting comparison between numbers that are within or beyond the curriculum-defined knowledge. As a reference, we also examined a smaller group of 4th-grade children.

Last, we examined which kind of knowledge supports the children’s learning to read numbers. In line with our hypothesis that the main challenge is syntactic, we assessed the children’s syntactic-verbal and syntactic-conceptual knowledge, and we examined whether either type of knowledge contributes to their ability to apply the syntactic rules correctly when they read numbers. If it does, we should expect a negative correlation between the corresponding syntactic knowledge task and the syntactic errors in the number reading task.

## Methods

### Participants

The participants were 97 third-grade children (50 females) aged 8;10 (8 years 10 months), SD = 0;5, and 30 fourth-grade children (16 females) aged 9;11, SD = 0;3. One additional child was excluded for responding “I don’t know” to almost all stimuli in the number reading task. They attended standard, non-religious, Hebrew-speaking elementary schools in Israel, and they had no reported learning or attention disorders. Their native tongue was Hebrew. They were recruited via social networks. All children and their parents gave informed consent. The study was approved by the Tel Aviv University Institutional Review Board.

The sample size was set to be similar to another study that aimed to capture individual differences in number reading (Handelsman & Dotan, 2023). We also verified that it would be sufficiently large to detect the expected differences between the main types of errors, syntactic and non-syntactic (defined below). A G*Power analysis with *dz* = 0.5, alpha = 0.05, and power = 0.95, indicated a minimum of 45 participants.

The tasks were administered individually in one or two online video meetings. The participants could quit mid-task, and some did (they could still continue to the next task), but we encouraged them to keep on as long as they could. All 97 third graders performed the number reading task. The digit-in-position task was performed by 96 third graders, and the grammaticality decision task was performed by 37 third graders.

In supplementary material (10.17605/osf.io/S8397), we provide each child’s detailed responses to each task and stimulus and the per-child results of some analyses.

### Number reading task

#### Stimuli and procedure

In each trial, the participant read aloud in Hebrew a multi-digit number, presented as a digit string with no comma separator (10 numbers on screen at a time). Most participants read 115 numbers, and 14 of the 3rd graders read a different list of 105 numbers (this was just for technical reasons; the results were essentially the same when excluding these participants). All stimulus lists had similar characteristics: The numbers had 2–5 digits; about 60 numbers included the digit 0, which creates syntactic irregularity and therefore syntactic difficulty; and about 20 numbers included 1 in the tens position, yielding a teen word. In Additional file 1: Section S2, we provide the full list of stimuli and more detailed information about their characteristics. The numbers were shuffled randomly (same order for all participants), to discourage perseveration-based strategies. To ensure that the item-based analyses will be informative even if a participant does not complete the task, we verified that the first half of the list includes sufficient representation of items of different lengths and different syntactic structures (see details in Additional file 1: Section S2). Moreover, specifically for the analysis of error types (described in the next section), most items allow for most of the error types, so reading fewer items should not significantly bias the results.

In all lists, there were 5 training trials, which were not analyzed. Errors were corrected only in these training trials.

#### Error classification

Common models of number reading describe multi-digit numbers as involving three main types of information, which are handled by separate cognitive processes: the identity of each digit or number words, their relative order, and the number’s syntactic structure (Cipolotti & Butterworth, 1995; Cohen & Dehaene, 1991; Dotan & Friedmann, 2018; McCloskey, 1992). The syntactic structure is defined concretely as the *number word frame* (Cohen & Dehaene, 1991; Dotan & Friedmann, 2018)—the series of lexical classes (e.g., ones, tens, teens) and decimal words ("thousand") in the number (see Additional file 1: Section S1 for a detailed description of the Hebrew number system). Conforming to this distinction, we classified the errors into 3 types. A digit identity error is a substitution of one digit by another (e.g., 23 → 24) or not knowing a digit (23 → *twenty-something*). Syntactic errors are violations of the number’s syntactic structure, i.e., when the participant produced an incorrect number word frame. For example, reading 203 as "two thousand and three," "two hundred thirty," "two hundred thirteen," "two hundred zero three," etc. A digit order error is a change in the relative order of two digits (e.g., 23 → 32 or *thirty-something*). Note that transpositions of 0 with a non-0 digit (e.g., 320 → 302) could formally be classified either as an order error or as a syntactic error. We classified such errors as syntactic. This classification conforms to error classification in previous studies (Cohen et al., 2000; Dotan & Friedmann, 2018; Lochy et al., 2004; Moura et al., 2013; Noël & Seron, 1993), and it also has good theoretical justification, because the order of non-0 digits and the digit 0 is handled by separate cognitive mechanisms (Dotan & Friedmann, 2018; Dotan et al., 2021; Friedmann et al., 2010), presumably due to the syntactic particularity of the digit 0. Still, we verified that the error type analyses were essentially the same when classifying these cases as order errors.

## Results

### Number reading: general performance patterns

**Fig. 1:**
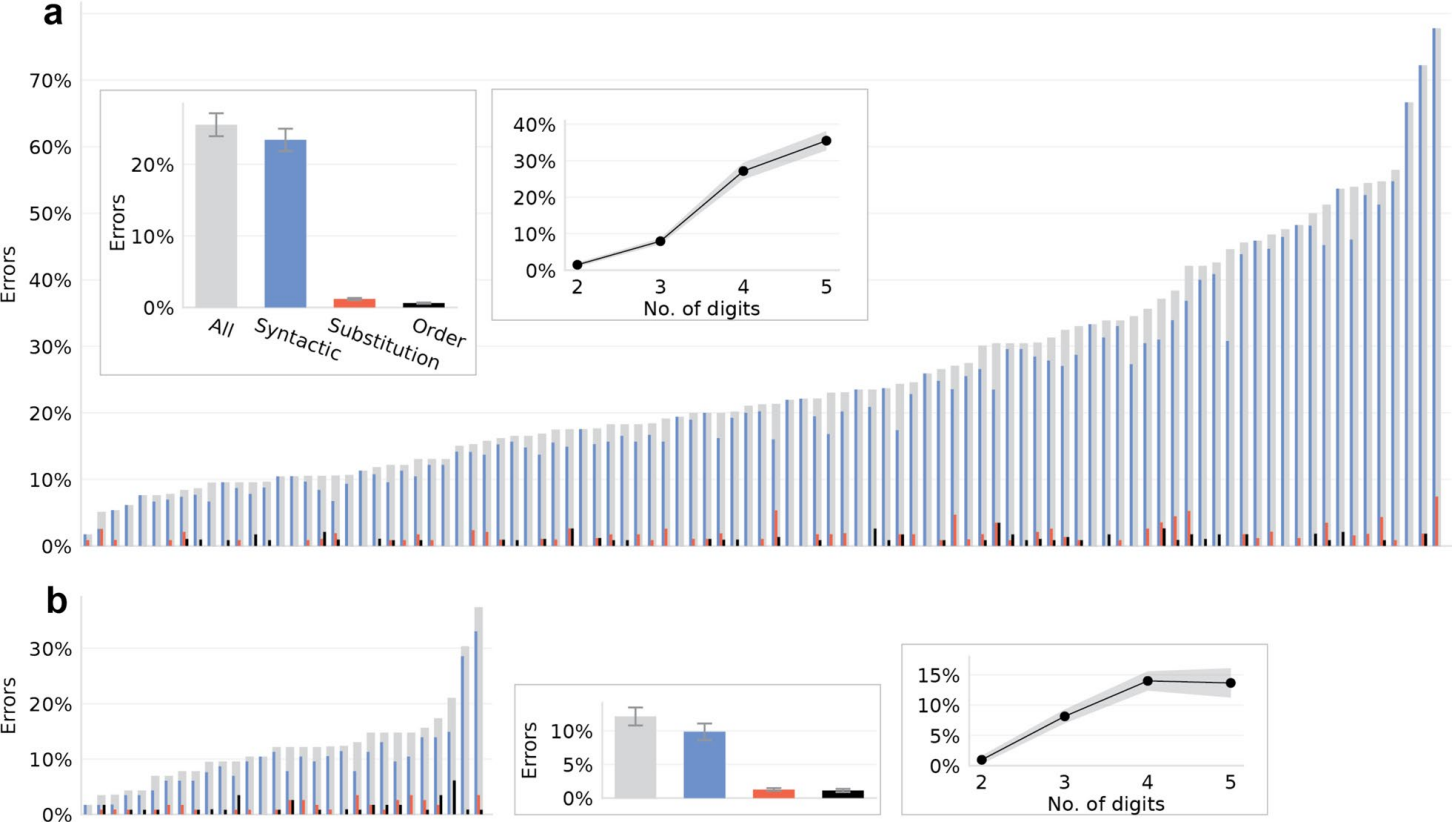
Error rates for **a** 3rd-grade and **b** 4th-grade children in reading aloud numbers with 2–5 digits. The thin bars show each child’s error rates (gray = overall, colored = specific error types, classification detailed in section “Error classification”). The insets show the mean rate of errors of each type, and the effect of number length on the overall error rate. Error bars/shaded area show one standard error of the per-participant means

Of the 97 third graders, 38 children read all items, 63 read more than 100 items, and 6 read 55 items. The average error rate was 25.3% (SD = 15.7%; Fig. 1a). A split-half test confirmed the task’s ability to evaluate individual differences between children: When dividing randomly each child’s items into two halves, the correlation between the error rates in the two halves was high (repeating this 1000 times: *r* = 0.76–0.91, mean = 0.85, SD = 0.02, all *p* < 0.001). Unsurprisingly, the fourth-grade children had fewer errors (Fig. 1b; mean = 12.0%, SD = 7.5%; unpaired *t*(125) = 4.42, one-tailed *p* < 0.001), and so did adults who read a slightly harder list of numbers (mean = 6.7% errors, SD = 4.3, Handelsman & Dotan, 2023).

The variance between the children was high: The top decile had 7.0% errors on average, not much more than adults who read slightly harder numbers (Handelsman & Dotan, 2023), and 22/97 children had fewer errors than the 4th-grade median (12.2%), but the bottom decile had 59.0% errors. This variability was not driven by the children’s age and was almost unaffected by the time in the academic year (see supplementary material).

### The origin of difficulty in number reading: syntax

An analysis of the children’s error types showed that processing the number syntax was a source of difficulty: Most of their errors were syntactic (Fig. 1; detailed error classification in Additional file 1: Table S4), both in the 3rd grade (syntactic vs. non-syntactic errors: paired *t*(96) = 14.1, one-tailed *p* < 0.001) and in the 4th grade (paired *t*(29) = 6.2, one-tailed *p* < 0.001). Even for individual participants, 125/127 children had more syntactic than non-syntactic errors, with a significant difference (chi-square one-tailed *p* ≤ 0.05) for 111/127 of them.

Another, more-anecdotal finding indicating that syntax is hard, was that in some cases we observed morphological errors in morphologically irregular numbers. For example, the number 2,000 in Hebrew has an irregular structure. In Hebrew 4-digit numbers, the thousand digit is translated into a single word in which the stem is an inflection of the corresponding unit word and the suffix is /talafim/, literally "thousands." For example, 3 is /shalosh/ and 3,000 is /shlosh-talafim/. The word for 2,000 is irregular: It is /alpaim/—a morphological combination of the word /elef/ (meaning 1,000) and the morphological suffix /aim/ (meaning "a pair of"). Several children read 2,000 incorrectly, often making a regularization error by replacing the irregular suffix /aim/ with the regular suffix /talafim/. In some cases, they combined the incorrect suffix with the correct stem /elef/ (resulting, for example, in /alpa-talafim/); more commonly, they regularized the stem too, by using a variant of the unit word "two" (e.g., /shnei/, resulting in /shne-talafim/).

**Fig. 2:**
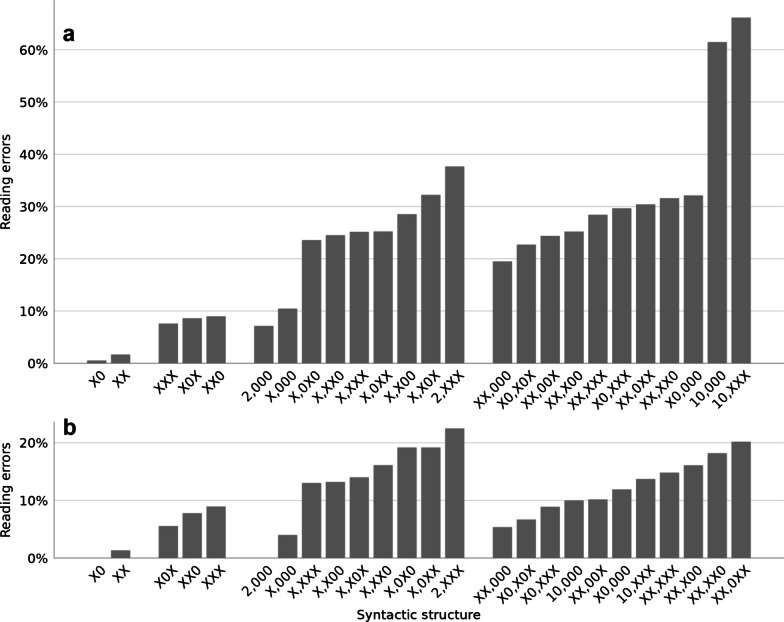
Error rates of the **a** 3rd graders and **b** 4th graders in reading aloud numbers with different syntactic structures, grouped by the number of digits. **a** In the third grade, the highest error rates were in the numbers with irregular syntactic structures (2,xxx and 10,xxx). Numerically, these numbers were not larger but smaller than other numbers with the same number of digits, supporting the idea that the degree of difficulty was affected by syntactic irregularity more than by the number size. **b** In the 4th grade, the error rates in the ten thousand (10,xxx) structure became lower, indicating that by this age the children have already learned this specific structure

We next contrasted syntax against numerical magnitude as potential origins of difficulty when reading numbers. Two findings recognize syntax as the crux of difficulty. First, in each number range (4 digits, 5 digits), the hardest numbers were not the largest ones but the smallest ones (Fig. 2). Specifically, the third graders made more errors in numbers starting with “two thousand,” i.e., 2000–2999, than in 3000–9999 (paired *t*(96) = 4.0, 1-tailed *p* < 0.001); and more errors in numbers starting with “ten thousand,” i.e., 10,000–10,999, than in 11,000–99,999 (paired *t*(96) = 8.7, one-tailed *p* < 0.001). These findings are opposite to the magnitude-driven-difficulty hypothesis. The simplest explanation is that the main origin of the difficulty was not numerical magnitude but syntactic irregularity, because the syntactic-verbal structures “two thousand” and “ten thousand” are irregular in Hebrew. The verbal-morphological form of 2000 is different from that of 3000–9000, as explained above, and similarly, the verbal-morphological form of 10,000 is different from that of 11,000–19,000 (see Additional file 1: for a detailed description of the Hebrew number syntax). An anecdotal finding in line with this idea was that two children sometimes confused the two irregular structures, reading a 10,xxx number as 2,xxx (e.g., 10,300 as “two thousand three hundred”). Such an error may appear strange because there seems to be no phonological, morphological, syntactic, or magnitude similarity between 2,xxx and 10,xxx, however, the error makes a lot of sense if the children’s internal representation classified both cases as “a syntactic irregularity.”

Second, to directly contrast the effects of magnitude and syntax across all items, we used a logistic linear mixed model with accuracy as the binary dependent variable, participant as a random factor, and two sets of within-participant factors. *Magnitude* was a single factor, defined as log(target). *Syntax* was a set of 4 factors reflecting the number’s syntactic-verbal structure: the number of digits; the positions of 0; the positions of 1, unless in a unit digit where it causes no syntactic irregularity; and the presence of 2 in the hundred or thousand positions. We excluded teen numbers (with them the model did not converge) and numbers with 3 or fewer digits. To assess significance, we used a log-likelihood ratio test and compared the full model to a model in which either the magnitude factor or the 4 syntactic factors were removed. Both magnitude (*χ*^2^(1) = 5.8, *p* = 0.02) and syntax (*χ*^2^(9) = 473.0, *p* < 0.001) had significant effects, but the stronger effect was that of syntax (full details in supplementary materials Table S5).

In the 4th grade (Fig. 2b), the effect of syntactic regularity was smaller: The 2,xxx numbers were still somewhat harder than the larger 4-digit numbers (22.2% vs. 13.8%, *t*(29) = 1.69, 1-tailed *p* = 0.05), but the 10,xxx numbers were no longer the hardest 5-digit numbers, suggesting that by the 4th grade, children already overcome the challenge imposed by the 10,xxx syntactic structure, and to a certain extent, also the challenge imposed by the 2,xxx syntactic structure.

### There is no single order for learning the different syntactic structures

**Fig. 3:**
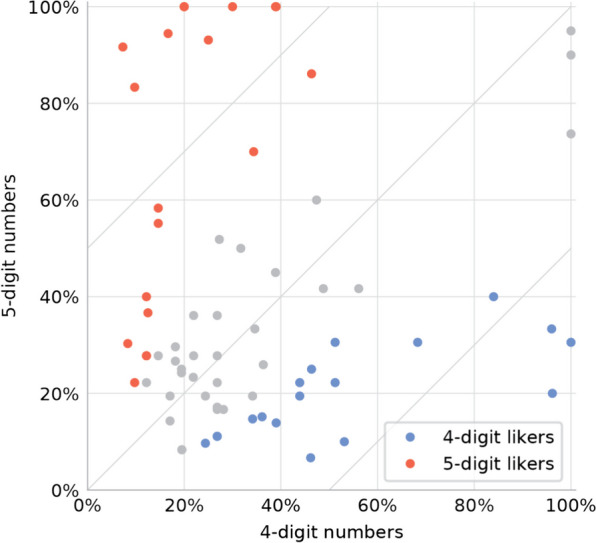
The error rates of each 3rd grader in reading numbers with 4 digits versus 5 digits (excluding the children with fewer than 10 errors and the items with the irregular syntactic structures: 2,xxx and 10,xxx). Colored dots indicate the 35/68 children whose error rate in numbers of one length was significantly higher than in the other

We next examined whether all children follow the same progression pathway when they learn to read numbers. Given the findings above, that the main challenge is syntactic, we compared numbers with two specific syntactic structures: 4-digit numbers and 5-digit numbers. Unlike English, in Hebrew 4-digit and 5-digit numbers have completely different syntactic structures, i.e., it is not the case that the syntax of 5-digit numbers “extends” that of 4-digit numbers (see Additional file 1: for a detailed description of Hebrew number syntax). If one structure is intrinsically harder than the other, we expect to find many children who make more errors in the harder structure, and fewer children who make more errors in the simpler structure. Contrary to this prediction, 51/97 children performed better in 4-digit numbers and 48 performed better in 5-digit numbers (Fig. 3). In this analysis, and in the remainder of this section, we excluded the numbers with very hard syntax (the irregular ones: 2,xxx and 10,xxx) or very easy syntax (fully round numbers, i.e., those with a single non-0 digit).

This finding—that about half of the children were better at 4-digit numbers and half at 5-digit numbers—could be interpreted in two ways. The trivial explanation is that the difference is random, i.e., that errors were distributed randomly between 4-digit and 5-digit numbers. A more interesting explanation, however, is that 4-digit can be genuinely harder or easier than 5-digit numbers, but the direction of this number length effect is different for different children. In other words, some children are genuinely better at reading 5-digit numbers better than at reading 4-digit numbers ("5-digit likers"), and other children are the opposite ("4-digit likers"), and when examining the group as a whole, these two effects cancel each other. Note that being a "5-digit liker" does not mean that a child just randomly happened to make fewer errors in 5-digit numbers than in 4-digit numbers; it means that there is a real principled reason for which that child made fewer errors in 5-digit numbers, i.e., the child has better ability to read aloud 5-digit numbers than 4-digit numbers. Thus, for a child to be defined as a 5-digit liker, it is not enough that he makes fewer errors in 5-digit numbers, but the difference between 4-digit and 5-digit numbers must be larger than the difference expected by chance.

#### Some children are 4-digit likers and others are 5-digit likers

To examine the idea presented above, we ran a statistical analysis whose goal was to determine whether there exist any 4-digit likers or 5-digit likers—children whose performance in numbers of one length is better than their performance in the other length, relative to the chance-level expectations. Note that this is not a standard condition-comparison analysis, because our goal was not to compare the performance between 4-digit numbers and 5-digit numbers at the group level. Rather, our goal was to examine whether there exist children who significantly deviate from any putative group-level difference between 4-digit and 5-digit numbers, and to test this separately for each direction (4-digit likers, 5-digit likers). Note also that the analysis did not aim to show who the 4- or 5-digit likers were: only to examine whether such children exist. Thus, the analysis aimed to assign only two *p*-values to the whole group: one for the existence of 4-digit likers and another for the existence of 5-digit likers.

##### Methods

We describe the analysis that assessed the existence of 4-digit likers; the analysis for 5-digit likers was the same, in the opposite direction. The specific goal of this analysis was to examine whether there exist any children who make more errors in 4-digit numbers than in 5-digit numbers, and whose "preference" for 4-digit numbers exceeds the chance level. Importantly, note that even by chance, we do not expect the children’s performance to be purely random. We defined "chance" as the conjunction of 3 criteria: (1) Irrespective of number length, some children might be better readers than others. (2) At the group level, irrespective of a particular child’s ability, 4-digit numbers may be easier or harder than 5-digit numbers. (3) Critically, a particular child’s performance would not be biased toward 4-digit numbers or 5-digit numbers any more than predicted by group-level preference. In other words, a child’s performance in a particular number may be affected by the child’s overall level and by the overall difficulty of numbers of that length, but these two factors do not interact with each other. In contrast, if some children are 4-digit likers, the two factors will interact such that some children will read 4-digit numbers better than the conjoint prediction of the child’s overall ability and the overall difficulty of 4-digit numbers.

To examine this prediction, we used a bootstrap procedure: We generated 50,000 random datasets according to the above definition of randomness, and we compared the dependent variable (the number of 4-digit likers) between the observed data and the random datasets.

*Dependent variable computation* The dependent variable, computed for the observed data and for each random dataset, was the number of 4-digit likers. For this sake, a participant was defined as a 4-digit liker if their error rate in 4-digit numbers was lower by at least 33% than their error rate in 5-digit numbers (similar results were obtained when the criterion was changed to participants who have at least twice as many errors in 5 digits than in 4 digits).

*Generating a random dataset* Each of the 50,000 random datasets specified how many errors each child would randomly make in each number length. Per the 3 randomness criteria defined above, each random dataset maintained the overall error rate of each child, pooled over the two number lengths; and the overall error rate in each number length, pooled over all children. We randomized only the Child × Length interaction, i.e., how each specific child’s errors are distributed between the two number lengths.

In the following explanation, the term *4-digit error percentage* denotes the average percentage of 4-digit numbers out of all errors in a given dataset, i.e., the average of the per-child $\frac{{\#\;{\text{of 4-digit errors}}}}{{\left( {\# \;{\text{of 4-digit errors}}} \right) + \left( {\# \;{\text{of 5-digit errors}}} \right)}}$. Before generating the random datasets, we computed the 4-digit error percentage in the observed data—hereby, *P*_4Global_. Then, to generate a particular random dataset, we randomized the errors by dividing each child’s observed errors randomly between the 4- and 5-digit numbers using binomial distribution with probability *P*_4Global_. This division brought the randomized 4-digit error percentage to be close to *P*_4Global_. We then multiplied each child’s number of errors in 4-digit numbers by the fixed factor (same for all children) that would bring the group’s 4-digit error percentage to be precisely *P*_4Global_ (the number of errors in 5-digit numbers was changed correspondingly to maintain the child’s overall number of errors).

*P-value computation* As stated above, number of 4-digit likers was computed for the observed data and for each random dataset. The *p* value for the existence of 4-digit likers was defined as the rate of random datasets that yielded at least as many 4-digit likers as in the observed data.

Essentially, the process above compares the number 4-digit likers to the theoretical H_0_ distribution derived from the assumption that each participant’s errors are divided between 4-digit numbers and 5-digit numbers randomly, i.e., according to a binomial distribution with mean = *P*_4global_. We verified that similar results were obtained when the value of *P*_4global_ (or *P*_5global_ in the analysis of 5-digit likers) was set to a fixed value of 50% rather than according to the real data.

##### Results

We analyzed only the 69 children who had at least 10 errors, because with a small number of errors the bootstrap process may overestimate the likelihood of being a 4-digit liker or a 5-digit liker (similar results were obtained for a 15-error threshold). The analysis revealed a significant double dissociation between the two number lengths: The number of 5-digit likers was significantly higher than chance, and so was the number of 4-digit likers (*p* < 0.001 for both).

#### How many 4-digit likers and 5-digit likers are there?

We next examined, for each participant who had a higher error rate in one number length (4 or 5 digits) than in the other, whether this difference was significant—i.e., whether that specific participant was a "4-digit liker" or a "5-digit liker." Thus, the analysis assigned a separate *p* value to each participant, which estimates the likelihood that this child is a 4- or 5-digit liker. The goal was to estimate the prevalence of the phenomenon, i.e., how many children read numbers of one length better than numbers of the other length.

##### Methods

The analysis was a bootstrap process similar to the group-level analysis described above. It was run for all participants together, but it computed a separate p-value for each participant.

We first computed, for each participant, the number length effect size: the difference between the error rate in 4-digit and the error rate in 5-digit numbers. We then generated 50,000 random datasets precisely as we did in the group-level bootstrap analysis. The *p*-value of each child was defined as the rate of random datasets in which that child’s number length effect was in the same direction as in the observed data and at least as large.

##### Results

A significant difference between the conditions was found for 37/69 children—i.e., the child-level dissociation between the two number lengths was not a rare phenomenon but a common one, covering more than half of the children. Interestingly, children were as likely to find the larger numbers (5 digits) easier as they were to find the smaller ones (4 digits) easier: 18 children were 4-digit likers and 19 were 5-digit likers (Fig. 3).

#### Conclusion from the existence of 4- and 5-digit likers

The existence of 4-digit likers and 5-digit likers shows that the syntactic structures of 4-digit numbers and 5-digit numbers do not depend on each other. Rather, each syntactic structure can be learned first, independently of the other. Moreover, neither structure is intrinsically harder to start with than the other—children were as likely to prefer 4-digit numbers or 5-digit numbers. Apparently, in the third grade, being in this intermediate developmental stage, in which a child masters one number length better than the other, is not a rare phenomenon, nor is this developmental stage very short, because more than half of the children we examined had a significant preference for one number length over the other.

### Learning to read numbers is related to syntactic-verbal knowledge, less to syntactic-conceptual knowledge

The last question we examined concerned the type of knowledge needed to learn number syntax: syntactic-conceptual knowledge, syntactic-verbal knowledge, or both. Our study was cross-sectional, and we acknowledge that such a design does not allow for drawing reliable causal conclusions, however, correlation patterns can still be informative about the possibility of causal relation. We reasoned that if a certain type of knowledge drives syntactic processing in number reading, then the performance in a task that taps this knowledge would correlate with the syntactic error rate in number reading.

#### Syntactic-conceptual knowledge

To tap syntactic-conceptual knowledge of the decimal number system as a symbolic system, we used a task, common in schools, of naming particular digits in a number. In each trial, the participant saw a multi-digit number and was asked to say the digit in a particular decimal position: units, decades, hundreds, thousands, or ten-thousands (e.g., “What is the decade digit in 2,345?”). This task taps conceptual knowledge about the decimal system structure—e.g., that decimal position matters, that it is defined relative to the number’s right end, and the decimal class names (units, decades, etc.). There were 3, 5, and 5 items with 3, 4, and 5 digits, respectively (13 items in total). Each child completed at least 8 items (13, 18, and 64 children with 8, 10, and 13 items, respectively), except one child who completed only 4 items and was excluded. We examined the correlation between the error rate this task and the syntactic error rate in the number reading task, from which we considered only the numbers with at least 3 digits.

**Fig. 4:**
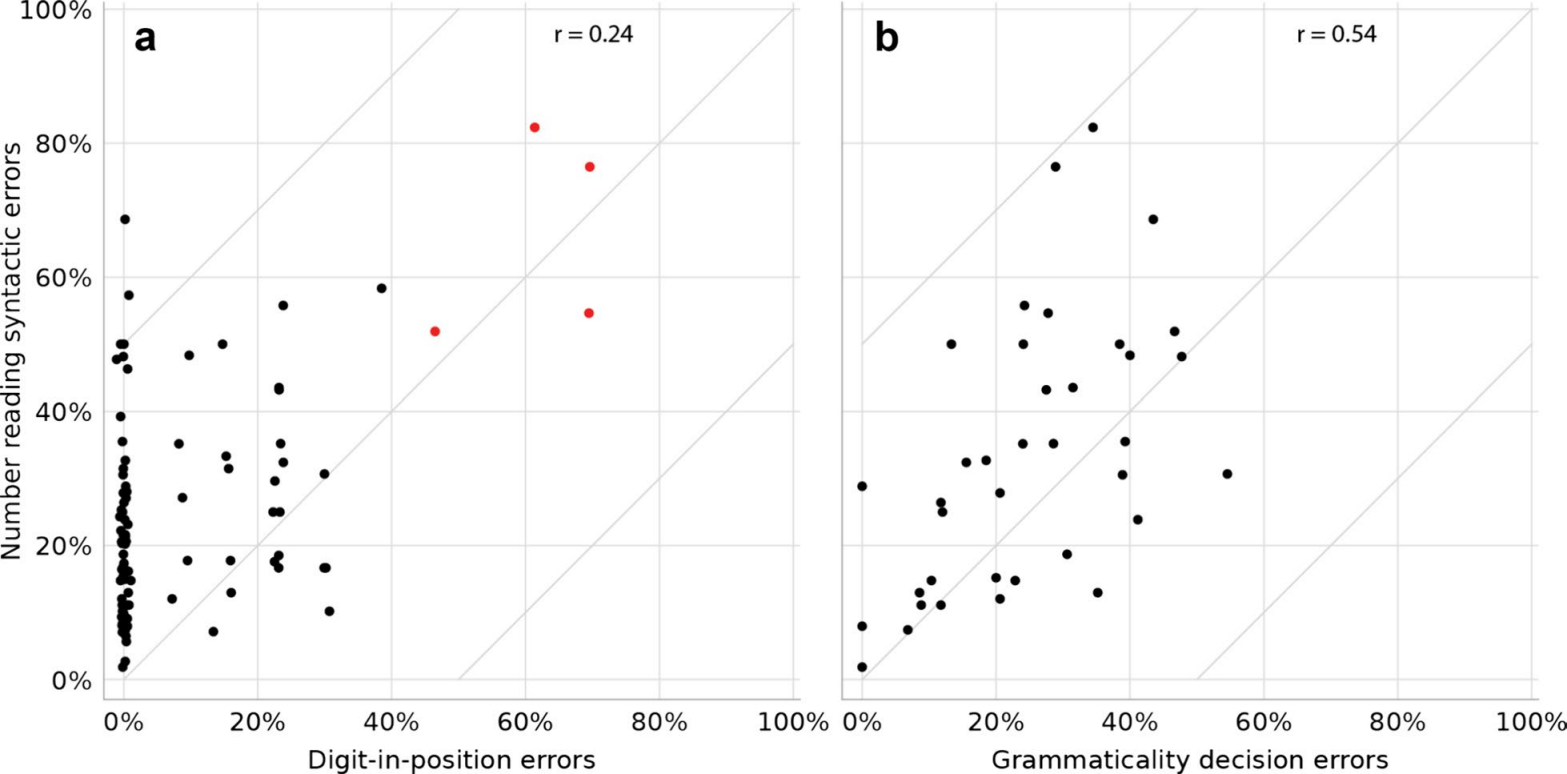
The syntactic error rate in number reading versus the error rates in **a** the digit-in-position task (e.g., “what is the hundred digit in 2,975?”; n = 95 children), which taps knowledge of the decimal system structure; and **b** the grammaticality decision task (“is ‘thirty-two’ grammatical?”, “is ‘fifty-sixty’ grammatical?”, etc.; *n* = 37 children), which taps knowledge of verbal-syntactic rules. Each dot represents one child. In **a**, minor horizontal jitter (< 1.5%) was added for visual clarity, and the correlation was computed without the 4 participants who were outliers in the digit-in-position task (red dots). Grammaticality decision was a good predictor of number reading, but the digit-in-position task was not

The digit-in-position task turned out to be a poor predictor of syntactic errors in number reading. First, although the correlation between the two tasks was *r* = 0.53 (one-tailed *p* < 0.001, Fig. 4a), this correlation was largely driven by merely 4 children who performed extremely poorly in the digit-in-position task (more than 38.5% errors; this outlier threshold was computed as the 75th percentile plus 150% the inter-quartile range). Without these 4 outliers (red dots in Fig. 4a), the correlation was significant but weak (*r* = 0.24, one-tailed *p* = 0.01). Second, many children performed at ceiling in the digit-in-position knowledge task but still had many reading errors (but the low correlation was not an artifact of this ceiling effect—it was still low even when we examined only the 27 children who had more than 7% errors in the digit-in-position task, *r* = 0.13). Third, the per-child number of syntactic errors in number reading differed significantly from the number expected if assuming that the number reading and digit-in-position tasks reflect the same skill (*χ*^2^(92) = 221.6, *p* < 0.001; we excluded 4 children with expected value < 5, but the results were similar when including them). To compute this expected number of errors, we defined each child’s *AvgErr* as the average between the error rate in the knowledge task and the syntactic error rate in the reading task, and we “redistributed” among the participants the total number of syntactic errors in number reading in proportion to the per-child *AvgErr*.

Still, although the digit-in-position task was a poor predictor of number reading when examined over all children, the very poor performers in the digit-in-position task also had many number reading errors. This pattern suggests that the syntactic-conceptual knowledge captured by the digit-in-position task may be necessary for number reading, but not sufficient.

#### Syntactic-verbal knowledge

To tap syntactic-verbal knowledge, we used a task in which participants judged whether sequences of number words were grammatical or not. English examples are “three thousand and five” (grammatical) or “forty thousand hundred” (ungrammatical). This task reflects the knowledge of specific syntactic rules of number reading. If the sequence was non-grammatical, the child was asked to fix it and say the correct number, but these corrections were not analyzed. There were 54 items (sequences); each child performed at least 23 items. We examined the correlation between the error rate this task and the syntactic error rate in the number reading task, from which we considered only the numbers with at least 3 digits.

Unlike the syntactic-conceptual task, the grammaticality decision task was a better predictor of syntactic errors in number reading (Fig. 4b). The correlation between the two tasks was high (*r* = 0.54, one-tailed *p* < 0.001) and it did not seem to be driven by a small subset of the children. Moreover, the per-child number of syntactic errors in number reading differed only slightly, even if still significantly, from the number of syntactic errors predicted by the assumption that both number reading and grammaticality decision reflect the same skill (*χ*^2^(34) = 51.8, *p* = 0.03; two children with expected value < 5 were excluded; the expected distribution was compared as in the previous section).

#### Comparing syntactic-verbal versus syntactic-conceptual knowledge

To directly compare the two tasks—digit-in-position and grammaticality decision—as predictors of number reading skills, we entered the error rates in the two tasks as predictors in a linear regression on the syntactic error rate in number reading. We included the 33 participants who performed all three tasks (excluding the 4 poor performers in the digit-in-position class). Grammaticality decision was a good predictor of number reading (*β* = 0.50, one-tailed *p* = 0.002), but digit-in-position was not (*β* = 0.18, one-tailed *p* = 0.13). To further confirm the differential contribution of each predictor, we ran a hierarchical regression and examined how each predictor contributes on top of the other. Grammaticality decision significantly improved the regression model on top of the digit-in-position task (Δ*R*^2^ = 0.22, *p* = 0.004), but the digit-in-position task did not have a significant contribution on top of grammaticality decision (Δ*R*^2^ = 0.03, *p* = 0.26).

These results confirm that the knowledge most relevant to number reading is the one captured by the grammaticality decision task, namely syntactic-verbal knowledge, and not the syntactic-conceptual knowledge captured by the digit-in-position task.

## Discussion

### Learning to read numbers is hard—and for some children it’s harder

The third-grade children had many errors in the number reading task (~ 25% on average). This is perhaps not surprising, because children at this age are still learning the decimal system, and we asked them to read numbers above 10,000, which they had not yet learned at school. In the fourth grade, number reading has already improved significantly—i.e., the third and fourth grades are critical stages in number reading development.

The variability among the third graders was high. Some children made almost no mistakes at all and performed nearly as well as adults, whereas other children made many mistakes, some even surpassing 70% errors. This high variability could not be explained by a simple factor such as the child’s age or the time during the academic year, and it is also unlikely to stem from dysnumeria, a learning disorder that disrupts number reading, because the estimated prevalence of dysnumeria in Hebrew (based on adults’ performance) is about 7% (Handelsman & Dotan, 2023). We conclude that the variability reflects normative differences in the typical development of number reading skills—differences that may originate in cognitive, educational, environmental, or other factors.

An important consequence of these findings is that it might be difficult to diagnose deviations from the typical development, namely dysnumeria, before the fourth grade. First, our findings indicate that many children at this age have not yet acquired fully the knowledge about how to read numbers, but to diagnose a child’s cognitive development we must assume that the child has the knowledge needed to perform the task at hand. Second, the large variability in typically developing children could make it difficult to detect statistical deviations, at least if we use numbers with 4 digits or more. Critically, assessment of dysnumeria might be ineffective if limited to shorter numbers, because reliable assessment might require examining the more complex syntactic structures, and these occur only in longer numbers.

### The origin of the difficulty is syntax

Two main findings indicate that the major origin of the difficulty in reading numbers is the difficulty imposed by syntactic processing or syntactic knowledge. First, most of the children’s errors were syntactic, and only a few errors were of other types (substitution, digit order). Second, the main factor that affected the error rates was syntactic complexity: Syntactically irregular numbers were harder than regular numbers. These findings replicate previous studies on number reading in children (Barrouillet et al., 2004; Moura et al., 2013; Power & Dal Martello, 1997; Seron & Fayol, 1994; Steiner et al., 2021; Van Rinsveld & Schiltz, 2016) and adults (Handelsman & Dotan, 2023) and extend them in two important respects. First, we showed that syntactic complexity affects the reading difficulty and that this effect is larger than that of magnitude. This is important because numerical magnitude is often considered to be a critical factor affecting the difficulty of processing a number—a problem-size effect, which is well documented in the context of calculation (Groen & Parkman, 1972; Zbrodoff & Logan, 2005), but as we saw here, it is not the main story in the context of number reading. Second, we observed the syntactic difficulty—more syntactic than non-syntactic errors—not only at the group level but also for almost each child, in a large group of 127 children. Number syntax was the main challenge for virtually everyone.

Our findings extend some previous studies also with respect to the specific type of syntactic errors observed. A well-known study that examined number reading in children is Power and Dal Martello (1997), who analyzed reading errors in second-grade Italian children. Similar to us, they too showed that the majority of errors were syntactic; however, the specific types of syntactic errors were different in the two studies. In Power and dal Martello’s data, a large portion of the errors were fragmentations of the number (e.g., reading 395 as “thirty-nine and five”), omission of the decimal words “hundred” (cento) and “thousand” (mille), or other errors in these decimal words. Such errors were rare in our data. We cannot point unequivocally to the reason for this difference between the two studies, but several reasons could be proposed. First, the children in Power and Dal Martello’s experiment were younger, and it is possible that the errors they made, in particular the fragmentation errors, reflect less mature strategies to cope with the syntactic difficulty. Second, in our experiment, the numbers were presented in mixed order, whereas Power and Dal Martello grouped them into blocks according to their syntactic structure. The blocked presentation, and the absence of syntactic variance within each block, may have encouraged the children to use more or other strategies. Finally, the morpho-syntactic structure of verbal numbers is different in Italian and Hebrew: In Italian, the decimal words “cento” and “mille” are separate words, similar to English. In contrast, in spoken Hebrew (albeit not in written Hebrew), the hundred class and, in 4-digit numbers, also the thousand class are not marked syntactically using a separate decimal word, but rather morphologically, by inflecting the word’s stem (similar to the -ty suffix for tens in English).

### The type of knowledge that supports number reading skills

The main task we used, reading numbers aloud, required the children to exhibit their ability to apply the syntactic rules in practice via a set of cognitive processes. We compared their number reading performance against two tasks that tapped other, more explicit levels of syntactic knowledge. The digit-in-position task, which taps syntactic-conceptual understanding of the decimal system, was a poor predictor of number reading, specifically of the syntactic error rate in number reading, although very bad performance in the digit-in-position task did predict a high rate of number reading errors. We propose that syntactic-conceptual knowledge may be critical for children to develop proficient number reading, however, it is not sufficient for proficient reading. Pedagogically, this is an important point, because at least in some education systems (e.g., Israel), digit-in-position and similar conceptual tasks seem to be the main kind of explicit training that children get about the symbolic base-10 system. If these tasks are not good facilitators of number reading skills, perhaps we should not be surprised that so many children and even adults struggle with reading numbers (Handelsman & Dotan, 2023).

In contrast, knowledge of syntactic-verbal rules, as measured via the grammaticality decision task, was a good predictor of number reading proficiency. This may suggest that syntactic-verbal knowledge facilitates reading proficiency. Note that such conclusions about the impact of knowledge on number reading performance should be taken with caution, because our design was cross-sectional, which does not allow for reliable conclusions about causality. Still, the idea that explicit teaching improves performance is not new, including in mathematics (Doabler & Fien, 2013).

Even if syntactic-verbal knowledge facilitates number reading skills, it remains an open question whether such knowledge is a sufficient condition for proficient reading. Presumably, the answer is not a simple “yes,” for at least two reasons. First, to obtain proficiency, knowledge of principles must typically be accompanied by practice. Indeed, while several researchers described the learning of syntactic-verbal rules as a critical stage, they also emphasized that this stage must be followed by an additional stage of automatizing the skill (Barrouillet et al., 2004; Power & Dal Martello, 1997). Second, at least in extreme cases, syntactic-verbal knowledge dissociates from reading skills: When a person has dysnumeria, a cognitive disorder that disrupts number reading, they make many reading errors, including syntactic errors (Cipolotti, 1995; Handelsman & Dotan, 2023; McCloskey et al., 1986; Noël & Seron, 1993), but often they can still explain how numbers should be read, and they even manage to read many of the stimuli correctly.

While our findings indicate that syntactic-conceptual and syntactic-verbal knowledge are separate, in no way do we propose that they are totally independent of each other. Quite the contrary—several studies indicate that the two are related. For example, the syntactic-verbal structure of the number system in a particular language seems to affect how speakers of that language learn the principles of the base-10 system (Fuson & Kwon, 1991; Miura et al., 1993; although see Saxton & Towse, 1998 for a complementary opinion). Moreover, syntactic-conceptual and syntactic-verbal knowledge are presumably related not only to each other, but also to other aspects of numbers and mathematics—e.g., the verbal-syntactic structure of numbers affects people’s way of performing calculations (Noël & Seron, 1997). An interesting open questions is how these different aspects of numbers and math—conceptual knowledge, verbal knowledge, and cognitive processes—interact during development and affect how children learn and achieve fluency.

### Syntactic knowledge is highly specific

The syntactic knowledge, a critical aspect of number reading skills, turned out to be highly specific, from two angles. One angle concerns the "level" of knowledge: As we saw above, syntactic-verbal knowledge is separate from syntactic-conceptual knowledge, and the former is related to number reading ability more than the latter. The second angle, which is orthogonal to the first, is that different specific syntactic structures are learned separately and relatively independently of each other. Two findings highlight this second point.

First, we observed a double dissociation between different syntactic structures: Some children had difficulty with 4-digit numbers and could read 5-digit numbers, and other children showed the opposite pattern. This double dissociation means that neither structure is a prerequisite for the other—in that sense, they are independent. Importantly, the double dissociation was not rare but a mainstream phenomenon, covering more than half of the children we examined. Second, we observed dissociations between different syntactic structures even within numbers of a given length: There were more errors in numbers with irregular syntactic structures ("two thousand" and "ten thousand") than in the regular numbers. The simplest explanation for these findings is that each specific syntactic structure requires specific learning. The children learned 4-digit numbers before 5-digit numbers or vice versa, and they learned the syntax of standard (regular) numbers before the “two thousand” and “ten thousand” irregularities. Each syntactic structure they learned did not suffice to support other structures.

A surprising finding, which we did not expect, was the extent of the double dissociation between 4-digit and 5-digit numbers. Half of the children showed dissociation, and critically, we observed no evidence that the shorter numbers (4 digits) were easier than the 5-digit numbers. The number of 4-digit likers, children who read 4-digit numbers significantly better than 5-digit numbers, was almost identical to the number of 5-digit likers. The Israeli math curriculum does not dictate that either of these syntactic structures should be taught explicitly at school (Israel Ministry of Education, 2006), and to the best of our knowledge, indeed schools do not teach these syntactic structures explicitly. We conclude that for these two syntactic structures, which do not intrinsically depend on each other, and perhaps for any such pair of syntactic structures, in the absence of formal schooling of syntactic-verbal structures, children are as likely to learn either structure first. The order of learning the structures might have to do with a child’s cognitive profile, but it could also be that the learning order is triggered by random environmental factors.

This highly specific structure, several syntactic rules that can be learned independently of each other, characterizes not only the syntactic-verbal knowledge. At the cognitive level too, the syntactic processing of multi-digit symbolic numbers is implemented by several separate cognitive processes (see review in Dotan & Brutmann, 2022), including several sub-processes within the verbal system. Presumably, conceptual knowledge too consists of several different aspects that could be learned separately. An open question is how this multitude of syntactic skills and processes is organized. One aspect of this question is that the verbal versus conceptual classification is not necessarily dichotomous—it might be more vague. For example, some inner representations might serve both syntactic-verbal and syntactic-conceptual skills—in fact, this was one of the central ideas in McCloskey’s (1992) number-processing model. A second aspect is that although the verbal versus conceptual distinction is a convenient and useful way to describe the different syntactic rules and skills, this does not necessarily entail that our knowledge is organized precisely this way at the cognitive/neurological level.

### Theoretical accounts of number syntax: knowledge versus cognitive processing

The idea presented above, that learning number syntax entails the acquisition of several separate syntactic rules, is central in developmental models of number reading (Barrouillet et al., 2004; Power & Dal Martello, 1997). These models are procedural and rule-based: They describe the series of operations and transformations needed to convert a number from one format to another, i.e., from a digit string to a series of number words and vice versa. In the 3-tier organization presented in Introduction—syntactic-conceptual knowledge, syntactic-verbal knowledge, and cognitive processing—such models fit the level of syntactic-verbal knowledge. In contrast, the idea of syntactic rules did not receive as much emphasis in cognitive "process models" (Cohen & Dehaene, 1991; Dotan & Friedmann, 2018; McCloskey, 1992). Such models typically emphasized the distinction between different types of cognitive processes: input versus output processes, the processing of verbal numbers versus the processing of digit strings, syntactic versus lexical processing, etc. Even when these "process models" describe the different types of syntactic processing (see review in Dotan & Brutmann, 2022), they often focus on the specific types of syntactic information (e.g., the number of digits as opposed to the positions of the digit 0) rather than on specific syntactic-verbal rules.

Historically, the "process models" of number reading were largely developed via studies with adult participants—neuropsychological studies with individuals with impaired number reading (e.g., Cipolotti, 1995; Cohen & Dehaene, 1991; Delazer & Bartha, 2001; McCloskey et al., 1986), and behavioral and brain imaging studies with typically developing adults (e.g., Dehaene et al., 2003; García-Orza & Perea, 2011). Correspondingly, these models do an excellent job at describing number reading in adults and explaining the performance patterns of individuals with specific cognitive deficits. However, process models seem inferior to rule-based models when the goal is to describe children’s development: In the present study, similar to other studies with typically developing children (Barrouillet et al., 2004; Power & Dal Martello, 1990, 1997), the results could be explained by the rule-based approach better than by the process models, because the dissociations we observed were between different syntactic rules (we did not report here our attempts to find dissociations between cognitive processes, but they were not as successful). Indeed, the existing process models remain largely silent about developmental questions. For example, they do not specify how and in which order the different cognitive processes develop, or whether children read numbers using the same cognitive processes as adults or different processes.

We hypothesize that the different focus of the two model types is not coincidental; rather, there is a genuine reason for which process models are better at describing adults’ performance whereas rule-based models are better at describing children’s performance. It may be the case that as typically developing children learn to read numbers, they learn the syntactic rules one after another, so during this period we can dissociate between the rules that they already learned and those they have not yet learned. Rule-based models can readily capture such dissociations. The knowledge of each new syntactic rule may be quickly “propagated” to all the relevant cognitive sub-processes, perhaps even to number reading processes and number writing processes as part of the same learning iteration. This leads to a relatively simultaneous development of the various syntactic cognitive processes, and consequently, dissociations between cognitive processes are scarce. The process models, which look for such dissociations, are therefore less informative in this case.

In contrast, the situation is presumably the opposite for adults with cognitive deficits. If a person is impaired in a particular cognitive process, this can be readily captured by a cognitive process model. If the person has already learned the syntactic rules, the impairment in the single cognitive process could affect several syntactic rules that require this process, so dissociations between rules would be scarce. The rule-based models, which look for such dissociations, are therefore less informative in this case.

An open question is how the rule-based models and the process models can be integrated in a unified theoretical framework. One idea, proposed by Dotan and Friedmann (2018), is that the different syntactic rules are implemented by different cognitive sub-processes only in specific processing stages. Almost trivially, such cognitive implementation of syntactic rules must be in verbal processes, because these rules typically reflect the syntactic properties of a particular language (Mark & Dowker, 2015; Van Rinsveld & Schiltz, 2016; Van Rinsveld et al., 2015). Nevertheless, at least in some cases, syntactic differences among languages affect also the cognitive processes that handle visual information (Dotan, 2023).

### Inter-language differences

The present study was in Hebrew, but our conclusions may apply also to languages in which the number system has similar syntactic characteristics. The situation might be different in other languages. For example, in languages in which the number system is fully regular, as is the case in several East-Asian languages, syntactic processing is presumably not as challenging. For example, in a study run in Hong Kong that compared children who learned numbers in Chinese/Cantonese versus English, the Chinese group exhibited greater proficiency in arithmetic tasks, especially at a young age. While these differences can be attributed to various factors, including cultural disparities in attitudes toward mathematics, at least one plausible explanation is that learning numbers in a regular language is easier than in an irregular language (Mark & Dowker, 2015).

What would our results look like had we run the study in a language with simpler number syntax, e.g., in China? We hypothesize that if syntax were simpler, its effect on number reading difficulty would be lower, and in turn, the relative contribution of other factors would be larger. For example, the relative contributions of syntax versus magnitude to reading difficulty might be different (but only if they are genuinely two separate factors; if the magnitude effect observed here was an artifact of syntax, e.g., because numbers with more digits are also more syntactically complex, switching to a syntactically simpler language may not make a big difference). Linguistic complexity could also affect the role of syntactic-verbal knowledge in learning to read numbers: With simpler syntax, this knowledge might be not as central, so the relation between syntactic-verbal knowledge and number reading may be weaker.

### Pedagogical implications

The present study was cross-sectional. We did not teach the children how to read numbers, and we certainly did not compare alternative teaching methods. Still, several of our findings entail potential implications for optimal pedagogical approaches—implications that could be examined in future intervention studies.

1. **Distinction between syntactic-conceptual and syntactic-verbal knowledge.** Tasks promoting conceptual understanding of the decimal system—e.g., knowledge of decimal class names (units, decades, etc.), the ability to identify the digits in particular decimal positions, and the place-value principle—are common in primary schools. While such knowledge is undeniably important (Mix et al., 2019), we saw that it is not sufficient to facilitate number reading skills. We therefore propose that schools should also teach syntactic-verbal knowledge.
  Such teaching could be done explicitly, by directly teaching specific rules of converting digit strings to verbal numbers (and vice versa), or implicitly, via continuous practice in tasks such as reading aloud and dictation of numbers. Both approaches—explicit and implicit—were shown to be effective in teaching syntactic skills, although each approach might have its limitations. Implicit learning of number reading was demonstrated in children, even with only a short passive exposure (Yuan et al., 2020), and in neural network models (Verguts & Fias, 2006; Yuan et al., 2020). Still, implicit learning might be limited. For example, Yuan et al.’s study used only a small number of syntactic rules, but difficulty might increase considerably as more rules are added—e.g., due to interference (Kliegl & Bäuml, 2021), or because extracting syntactic regularities implicitly might be very hard when the stimulus set includes numerous regularities. Moreover, Yuan et al.’s learning effect size was relatively small. This is not surprising given the small amount of exposure they provided, however, we may still ask whether obtaining fluency via implicit learning would require a lot of effort, and whether the amount of effort could be mitigated via explicit teaching.
  As for explicit teaching, to the best of our knowledge, this was not tested for number reading, but it was tested for learning the syntax of sentences in language. Explicit teaching of linguistic syntactic rules obtained better results than implicit teaching (Scott, 1990). Explicit teaching even helped remediate the grammatical skills of a child with a grammatical disorder (Specific Language Impairment, Levy & Friedmann, 2009). More generally, explicit teaching is considered by some to be critical for learning various aspects of math (Fuson & Briars, 1990). At the same time, explicit teaching too may have limitations: Presumably, obtaining fluency requires automatization which, in turn, requires sufficient practice. Moreover, implicit teaching methods might arguably be easier to incorporate in various educational activities, e.g., in games, which are an effective tool for learning (Vita-Barrull et al., 2024).
  In many domains, schools combine explicit teaching with practice and implicit learning. We propose that here too, they should do precisely this. Teaching syntactic rules explicitly could help children learn faster, as it saves the need to extract the syntactic rules implicitly from the stimuli. It can also provide non-automatic strategies as scaffolds that a child can use before automaticity is obtained. In parallel, a sufficient amount of practice could be critical to automatize the skill of converting digit strings to sequences of number words and vice versa.
2. **The degree of specificity within the syntactic-verbal knowledge.** We saw that the syntactic-verbal rules of number reading are largely independent, so we assume that they should be taught as such. Schools should teach each specific syntactic-verbal structure and each rule of converting a particular digit pattern into a particular verbal structure.
  An open question is whether number reading and number writing should be treated as a single skill or as two separate skills. We hypothesize that reading and writing build on similar procedural knowledge, even if the automatization of this knowledge, i.e., the ability to read and write numbers quickly, efficiently, and effortlessly, is implemented by largely separate cognitive processes for reading and writing (Cipolotti et al., 1994; Lochy et al., 2003; McCloskey et al., 1985). According to this view, syntactic-verbal knowledge should be taught as a single skill for reading and writing, but sufficient practice should be allowed both for reading and for writing.
  We further propose that the distinction between syntactic rules should be a guiding principle when teaching how to read and write numbers. We saw that the main challenge in handling a number lies in its syntactic structure, not in its magnitude, so it makes sense to group numbers according to syntactic rules rather than by quantity. For example, to teach 209 with 804 rather than with 210. Teachers may also group similar syntactic rules—e.g., discuss the similarity between 204 and 5,076, both of which include the zero irregularity, or the similarity between 476 and 8,259, both of which are fully regular (no zeros and teens).
3. **The order of learning the syntactic rules.** Some syntactic rules are easier than others, so it makes sense to teach them first. For example, in Hebrew, we should probably teach how to read 3000–9999 before teaching the “two thousand” irregularity, even if this contradicts the numerical order. In other cases, in which there appears to be no inherent “cognitive preference” for one syntactic structure over the other, the learning order should be established based on non-syntactic factors. For example, even if the syntactic structure of 4-digit numbers is about as difficult as that of 5-digit numbers, it seems to make sense to start with 4-digit numbers, because this aligns better with the numerical magnitude, and perhaps also with the difficulty imposed by performing arithmetic calculations on numbers of different lengths.

While some of these conclusions may appear simple, they are not trivial and not always implemented in the primary school curriculum. For example, in Israel, “number reading” is mentioned in the curriculum (Israel Ministry of Education, 2006), but only briefly and somewhat vaguely (except for the numbers up to 100). Likewise, the textbooks do not teach number reading explicitly (Center of Educational Technology, 2014; Koren et al., 2006; Luzon et al., 2006; Mevarech & Kremersky, 2016), they contain relatively little number reading/writing exercises, and the focus of these exercises is not the syntactic rules. To the best of our knowledge, in the classroom, there is little or no focus on number reading skills.

If the above conclusions do not dictate the existing pedagogical approaches for learning how to read numbers, what does? As far as we can see, it seems that curricula and textbooks reflect two implicit assumptions, both of which are at least partially incorrect. One assumption is that children will be able to generalize knowledge about the conceptual-quantitative aspect of the decimal system, e.g., the place-value principle and how to understand a multi-digit string as a quantity, into an ability to read and write numbers. This assumption is reflected by the focus on conceptual-quantitative exercises at school. While conceptual knowledge is clearly important for numerical literacy (Mix et al., 2019), we saw that such generalization, if it exists, is difficult or slow. The second assumption is that a number’s difficulty is largely driven by its magnitude. This assumption is reflected by the order of learning numbers, from small to large, and in the definition of learning goals in terms of numerical magnitude ("up to 1000," "up to 10,000," etc.). But as we saw, while magnitude is a critical factor in several tasks including calculation (Groen & Parkman, 1972), it is not the critical factor when learning to read numbers.

### Conclusion

The ability to read numbers may initially appear straightforward and trivial, but the present study joins a growing body of evidence indicating that this is not the case—certainly not for children, and not even for adults. These studies unequivocally implicate number syntax as the major source of difficulty in handling symbolic numbers. The present study extends this conclusion in several ways. We showed that when children learn to read numbers, the syntactic challenge is not merely to understand the decimal system at the conceptual level but also to learn several specific syntactic rules. We further showed that some syntactic rules are more difficult than others and that this differential difficulty does not always align with our intuition that larger numbers are harder. Last, we showed the large variance among children, in their overall number reading proficiency as well as in the order in which they acquired the different sub-skills of number reading; and we identified the third grade as a critical age in the development of number reading skills, at least for Hebrew speakers in Israel.

Numerical literacy has a fundamental role in our society, and the ability to read and write numbers is a central aspect of it. Reading numbers often serves as a student’s initial introduction to the world of numerical concepts, yet it can be complex and challenging for some. The conclusions presented here and in other studies call for a change in how schools teach the basics of numerical literacy. Specifically, we call to teach the challenging aspects of the symbolic number system, namely syntax, using a well-organized program that combines explicit teaching with practice. We believe this could considerably improve number reading and writing skills and help lay better foundations for numerical literacy. At the theoretical level, we argue that a unified theoretical framework for number reading and writing, which incorporates the distinction between different cognitive processes as well as the distinction between the specific syntactic rules, could be an important step toward a fuller understanding of how humans—both children and adults—handle symbolic numbers.

### Supplementary Information

Supplementary materials.

## Data Availability

The datasets generated and/or analyzed during the current study are available in the OSF repository, 10.17605/OSF.IO/S8397

## Footnotes

1 The syntax-magnitude correlation is not perfect. For example, the numbers 11–19 have an irregular syntactic structure which may make them more complex, and in French, the numbers between 70–79 and 90–99 (and perhaps also 80–89) also have an irregular structure. But for the most part, and particularly in larger numbers, which are the difficult ones, the correlation between syntactic complexity and magnitude is high.
