# Supplementary material for: Exploring the linguistic complexity of third-grade numerical literacy
Source: Cogn Res Princ Implic. 2024 Jul 18;9:48. doi: 10.1186/s41235-024-00575-5 (PMC11255179; doi:10.1186/s41235-024-00575-5)
Supplement: Supplementary file 1 — Supplementary materials. [file 41235_2024_575_MOESM1_ESM.pdf]

Supplementary material for

Exploring the linguistic complexity of third-grade numerical literacy

Ella Shalit and Dror Dotan

S1. The verbal number system in Hebrew

The syntax of Hebrew numbers is identical to English for numbers up to 100. Similar to English, there are number words for ones, teens, and tens (Table S1). The tens and teens words are derived from the ones words, in most cases with simple morphological modification (a minor phonological change and adding a suffix - /esre/ for teens, /im/ for tens). For example, 3 is /shalosh/, 30 is /shloshim/, and 13 is /shlosh-esre/.

Numbers larger than 100 are similar to English but with few differences:

1. **Hundreds words.** Unlike English, in which the hundreds are said as a ones word + the decimal word "hundred", in spoken Hebrew the hundreds are single words – a stem that reflects the digit, and a "hundreds" suffix (/-meot/). For example, the word for 3 is /shalosh/, and the word for 300 is /shloshmeot/. The English structure of "hundreds" with a 2-digit antecedent ("twenty-five hundred") is ungrammatical in Hebrew.
2. **Thousands.** The structure of 5- and 6-digit numbers is similar to English – a 2- or 3-digit number followed by the word "thousand" and another 1- to 3-digit number. The only difference is that the Hebrew word for "thousand" (/elef/) is phonologically quite different from "thousands" (/alafim/). In contrast, 4-digit numbers have a completely different syntactic structure, which is similar to the structure of hundreds words: "thousand" is not a separate word but a morphological affix, and for some numbers the stem of the word is slightly different phonologically from the corresponding ones word; for example, 3 is /shalosh/ and 3,000 is /shloshtalafim/.
3. **200 and 2,000.** The standard Hebrew form for a hundreds word is /X-meot/, and for a thousand word (in a 4-digit number) it is /X-talafim/. However, for 200 and 2,000 Hebrew does not use the standard suffixes but a single-word irregular form – 200 is /mataim/ and 2,000 is /alpaim/.
4. **10,000.** As explained above, Hebrew has a different syntactic structure for 4-digit numbers and for 5- and 6-digit numbers. The numbers between 10,000-10,999 are irregular because they do not use the 5-digit structure but the 4-digit structure – i.e., a morphological conjunction of the word "ten" with the "thousand" suffix. Thus, 10 is /eser/ and the 10,000 is /aseretalafim/.

The ones and teens words are inflected either to masculine or to feminine. When saying the number of objects, the number should agree with the object's masculinity or femininity (which is often arbitrary). For arbitrary numbers, which do not count objects (such as in our experiment),

Hebrew speakers usually use the masculine form for “one” (/exad/) and the feminine form for other numbers.

Table S1. The phonological forms of Hebrew number words (feminine).

	Ones	Teens	Tens	Hundreds	Thousands
1	axat	axatesre	eser	mea	elef
2	ʃtayim	ʃtemesre	esrim	mataim	alpaim
3	ʃaloʃ	ʃloʃesre	ʃloʃim	ʃloʃmeot	ʃloʃtalafim
4	arba	arbaesre	arbaim	arbameot	arbatalafim
5	xameʃ	xameʃesre	xamiʃim	xameʃmeot	xameʃtalafim
6	ʃeʃ	ʃeʃesre	ʃiʃim	ʃeʃmeot	ʃestalafim
7	ʃeva	ʃvaesre	ʃivim	ʃvameot	ʃvatalafim
8	ʃmone	ʃmonaesre	ʃmonim	ʃmonameot	ʃmonatalafim
9	teʃa	tʃaesre	tiʃim	tʃameot	tʃatalafim

S2. Experiment 1 stimuli

Following are the stimuli used in the number reading task. List #2 was administered to the 4 participants with IDs 1-4. List #3 was administered to the 10 participants with IDs 6-15. The remaining 83 participants read list #1.

Table S2. Characteristics of the stimuli in the number reading task (full list of stimuli in Table S1).

	List #1	List #2	List #3
No. of participants who read this list	83	10	4
No. of items	115	105	105
2-digit numbers	7	6	7
3-digit numbers	18	14	17
4-digit numbers	49	49	51
5-digit numbers	41	36	30
Numbers with 0	64	60	55
Teen numbers (1 as the decade digit)	20	19	18
Numbers with 2 as the hundred / thousand digit	6	7	7

List #1 *(main list – 83 participants)*

40,608	935	7,000	49,800	16	4,068
9,000	4,080	97,358	415	1,354	3,267
682	18	56,400	53,648	50,843	602
45,987	2,892	7,800	5,239	3,897	54,730
913	8,416	83,049	459	5,403	94,500
9,517	6,059	10,000	70,502	7,086	14,657
13,578	187	89,400	60	7,030	4,807
20,000	8,172	3,502	2,657	460	45,003
74,062	357	70,625	89,000	13	2,378
9,305	6,519	80,906	4,973	6,709	9,060
930	48,360	10,547	873	7,460	3,000
139	10,645	38,764	60,000	809	6,394
40	90,487	3,159	8,542	4,000	65,094
85,436	45	64,007	10,982	1,496	7,318
504	5,812	79,824	712	9,136	6,500
5,320	9,736	3,840	5,000	9,400	
8,032	145	357	9,570	10,743	
40,000	1,973	37,000	63	93,800	
7,384	95,027	87,590	16,745	890	
6,285	5,629	10,396	68,495	5,090	

List #2

14,963	16,745	5,840	89,400	459	3,000
3,897	890	80,906	930	50,843	9,736
13,578	70,502	7,000	7,086	6,500	73,068
9,400	2,378	64,007	6,394	2,895	357
18	504	8,500	45	40	7,514
93,800	74,065	187	8,036	918	7,030
3,267	90,487	48,360	419	7,450	174
60	20,000	5,316	45,003	95,067	7,318
65,094	10,891	6,709	9,518	4,068	3,976
5,639	7,800	10,000	38,764	14,657	9,000
4,807	6,285	604	70,645	3,159	609
5,000	460	79,864	5,239	40,608	45,987
10,738	6,374	2,657	935	5,390	6,080
9,136	10,547	53,648	68,495	9,705	97,358
4,000	40,000	4,983	5,090	13	9,570
87,390	10,642	873	2,000	5,403	
9,060	60,000	1,354	6,089	1,973	
3,709	10,743	5,814	63	85,436	

List #3

16	9,136	187	8,036	918	7,030
145	4,000	48,360	419	7,450	174
913	87,390	5,316	45,003	95,067	7,318
8,175	9,060	6,709	9,518	4,068	3,976
683	3,709	10,000	38,764	14,657	9,000
1,496	16,745	604	70,645	3,159	609
3,897	890	79,864	5,239	40,608	45,987
13,578	70,502	2,657	935	5,390	6,080
9,400	2,378	53,648	68,495	9,705	97,358
18	504	4,983	5,090	13	9,570
93,800	74,065	873	2,000	5,403	6,374
3,267	90,487	1,354	6,089	1,973	10,547
60	10,743	5,814	63	85,436	7,800
65,094	5,840	89,400	459	3,000	6,285
5,639	80,906	930	50,843	9,736	460
4,807	7,000	7,086	6,500	73,068	
5,000	64,007	6,394	2,895	357	
10,738	8,500	45	40	7,514	

In each list, the stimuli in the first and the second half of the list had relatively similar characteristics: distribution of the number of digits, and the presence of 0 (Table S3). Thus, reading only half of the list or more never resulted in an absence of some numbers.

Table S3. Characteristics of the stimuli in each half of each list.

	List 1		List 2		List 3	
	Half 1	Half 2	Half 1	Half 2	Half 1	Half 2
Numbers with 0	30/58	34/57	34/53	26/52	29/53	26/52
Number of items of each length:						
2 digits	3	4	2	4	3	4
3 digits	10	8	6	8	9	8
4 digits	22	27	22	27	24	27
5 digits	23	18	23	13	17	13

S3. Detailed error analysis

We classified the errors into substitution errors, order errors, and syntactic errors, as described in the main text (Section 2.3). The syntactic errors were further classified, as in previous studies with adults (Dotan & Friedmann, 2018; Handelsman & Dotan, 2023), into several subtypes:

Class error: producing the correct digit in an incorrect decimal class (one, tens, teens, hundreds, etc.). They consist of *decimal shifts* – cases in which the participant said the digit as if it was in a different decimal position (e.g., 230 → *two thousand and thirty*); and *teen errors* – substituting a teen word with a non-teen word (13 → *thirty*) or vice versa (30 → *thirteen*). Decimal shifts were further sub-divided into shifts in the first (leftmost) digit(s) of the number (2,305 → 20,305, 23,005, or 235), which is essentially starting to read the number as if it had a different number of digits than it had, and shifts in other positions (e.g., 2,305 → 2,035).

Thousand error: the word *thousand* was said incorrectly – omitted (e.g., 23,456 → *twenty-three*, *four hundred and fifty-six*), said in an incorrect position (234,500 → *two hundred thousand thirty-four and five hundred*), said more than once (e.g., 234,000 → *two hundred thousand and thirty-four thousand*), or distorted morphologically (in 4-digit numbers in spoken Hebrew, "thousand" is a morphological suffix rather than a separate word, but sometimes participants used the separate-word form. See supplementary material for details about the Hebrew number system).

Zero-substitution: a non-0 digit was substituted for 0 (e.g., 123 → 103) or vice versa.

Decomposition: the number was decomposed into a few separate verbal numbers (e.g., 2,345 → *twenty-three*, *forty-five*), sometimes due to producing the word "zero" (103 → *one hundred zero three*). We also considered it as a decomposition when the participant used the Hebrew 4-digit structure (in which the word thousand is not a separate word but a morphological affix) in a longer number, e.g., reading 54,000 as *fifty and fourthousand* or as *fivethousand and fourthousand* (the latter example is a combination of a first-digit decimal shift and a decomposition).

Like adults (Handelsman & Dotan, 2023), the main type of syntactic error was decimal shifts of the first digit (Table S4).

Table S4. Percentage of errors of each type in the number reading task.

		3rd grade[a]	4th grade[a]	Example for 23,040
Syntactic	All syntactic errors	23.4 (1.6)	9.9 (1.2)	
	First-digit decimal shifts	12.4 (1.1)	6.5 (1.2)	2,304 or 230,040
	Other decimal shifts	3.8 (0.3)	2.4 (0.3)	23,400; "twenty thirty thousand 40"
	Errors in the "thousand" word	5.0 (0.7)	0.9 (0.5)	"23 forty thousand"
	Decompositions	1.8 (0.7)	0.5 (0.3)	"23 zero 40"
Digit Order		0.6 (0.08)	1.1 (0.2)	32,040
Digit identity		1.7 (0.2)	1.8 (0.3)	25,040

[a] The error rates are the means of the per-participant averages, i.e., each participant had the same weight in this mean. The numbers in parentheses are the standard error of the per-participant averages. Classifications with less than 2% errors are not shown here.

[b] Example for the error of the given types when reading the digit string 23040. Note that the examples do not show all possible mistakes.

S4. Factors affecting number reading

4.1 3rd grade

We examined whether number reading was affected by Age, Sex, and the Time in Academic Year (number of days since September 1st, the beginning of the academic year), by entering these factors into a linear regression on the per-child error rates. There was no significant effect of Age ($\beta > 0$, i.e., opposite to the predicted direction, 2-tailed $p = .28$, Fig. S1). However, there was a marginally significant effect of Time in Year ($\beta = -0.17$, 1-tailed $p = .07$, Fig. S2) and a significant effect of Sex (male: 20.5% errors, female: 30.2%, standardized $\beta = 0.28$, one-tailed $p = .005$). This Sex effect replicates previous findings showing better arithmetic performance in boys (Han, 2019; Hill et al., 2016; Siregar et al., 2023). However, note that this demographic analysis might not be fully reliable because our sampling of participants was not systematic but snowball recruitment via social networks.

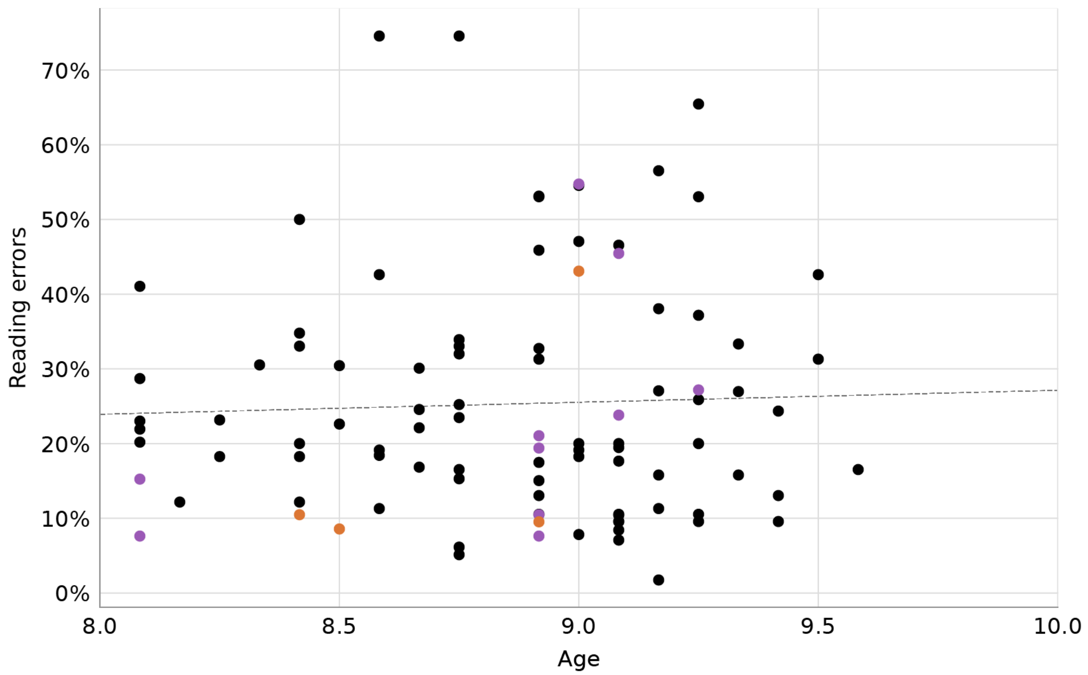

Fig. S1. Error rate for each child by their age. The dashed line shows the regression of the error rate against time. Black dots = list #1, orange = list #2, purple = list #3.

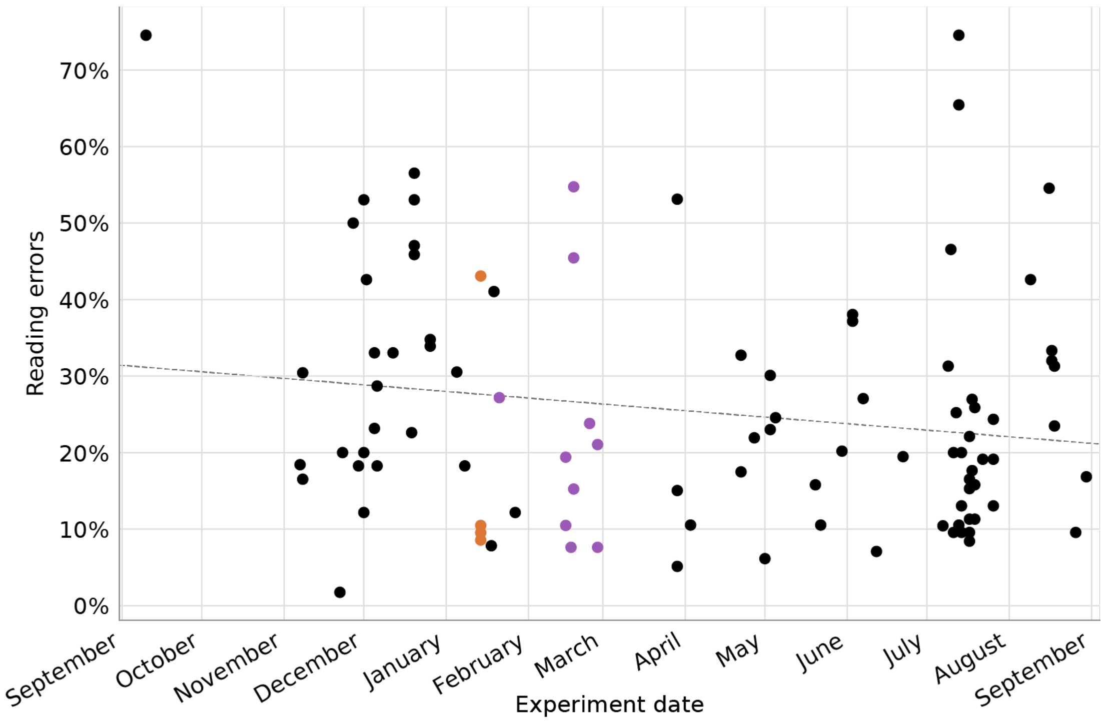

Fig. S2. Error rate for each child according to experimentation time during the school year (the school year starts on September 1st). Vertical gridlines indicate the first day of each month. The dashed line shows the regression of the error rate against time. Black dots = list #1, orange = list #2, purple = list #3.

4.2 4th grade

A linear regression on the error rates with Age, Gender, and the Time in Academic Year as predictors showed no significant effect of any of these factors.

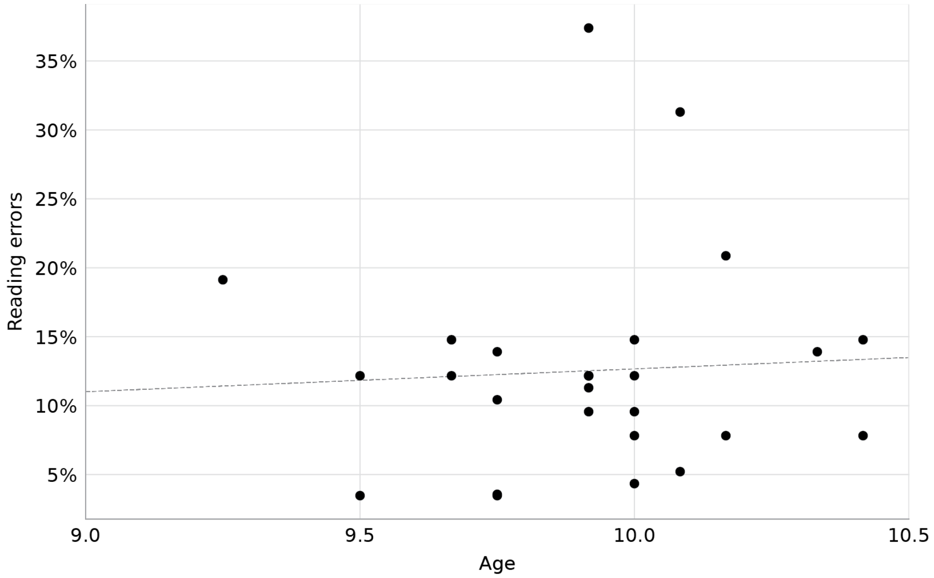

Fig. S3. Error rate for each child by their age. The dashed line shows the regression of the error rate against time.

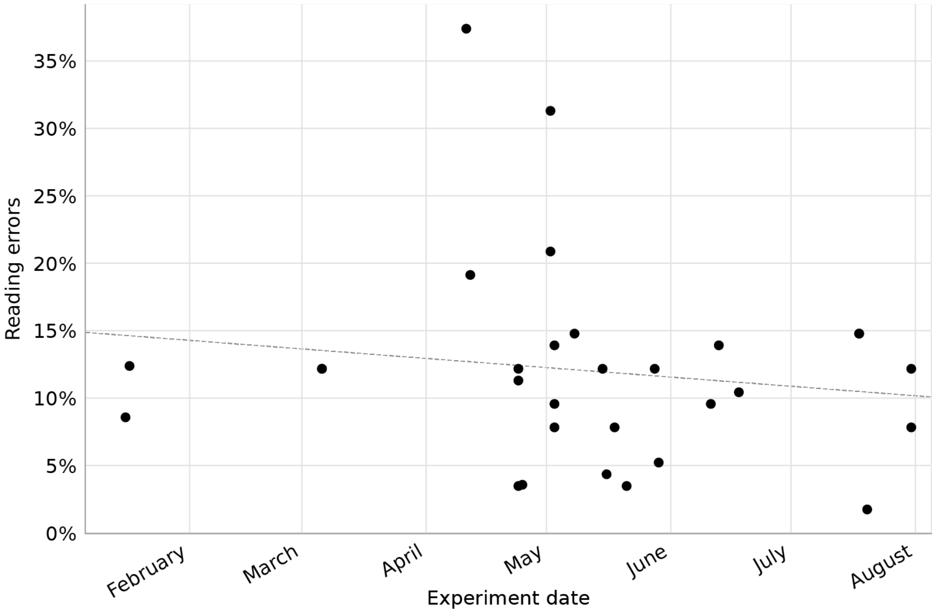

Fig. S4. Error rate for each child according to experimentation time during the school year (the school year starts on September 1st). Vertical gridlines indicate the first day of each month. The dashed line shows the regression of the error rate against time.

4.3 Detailed linear mixed model results (3rd grade)

Note that with respect to number syntax, the direction of the specific sub-factors whose effect was robust (odds ratio outside the 95% confidence interval) was in agreement with syntactic complexity: the 2,xxx and 10,xxx numbers were more difficult than others, and numbers with an empty right triplet (000) or with 1 as the hundreds digit were easier than others.

Table S5. Factors affecting accuracy in the number reading task – linear mixed model results. Both magnitude and syntactic structure had significant effects, but the effect of the syntactic structure was much stronger.

Predictor		Odds ratio	95% confidence interval[b]	Significance[c]
(Intercept)		2.28	1.69 – 3.06	
Magnitude	log(target)	0.75 ↓	0.60 – 0.95	$\chi^2(1) = 5.8$, $p = .02$
Syntax: Number length = 5		1.52	0.95 – 2.42	$\chi^2(9) = 473.0$, $p = 10^{-95}$
Syntax: 0 positions[a]	Right triplet = 000	1.79 ↑	1.46 – 2.20	
	At hundred	1.20	0.99 – 1.44	
	At end of triplet	1.15	0.96 – 1.38	
	At decade	1.05	0.86 – 1.27	
Syntax: 1 positions[a]	10,xxx	0.10 ↓	0.07 – 0.16	
	At hundred	1.94 ↑	1.36 – 2.78	
	At thousand	1.48	0.95 – 2.31	
Syntax: thousand digit = 2		0.49 ↓	0.35 – 0.68	
Random effects				
σ^2 within participants		3.29		
σ^2 between participants		1.13		

[a] In the linear mixed model, the baseline level for the "0 positions" sub-factor was numbers with no 0; for the "1 positions" sub-factor the baseline was numbers without 1.

[b] 95% confidence interval of the odds ratio. Factors with a significant effect on accuracy (1.0 is not included in the interval) in the positive direction are indicated by ↑, significant negative effects are indicated by ↓.

[c] Significance was tested by comparing the full model to a model in which the relevant factor/s were removed.

References

Dotan, D., & Friedmann, N. (2018). A cognitive model for multidigit number reading: Inferences from individuals with selective impairments. *Cortex*, *101*, 249–281. https://doi.org/10.1016/j.cortex.2017.10.025

Han, F. (2019). Self-Concept and Achievement in Math Among Australian Primary Students: Gender and Culture Issues. *Frontiers in Psychology*, *10*, 603. https://doi.org/10.3389/fpsyg.2019.00603

Handelsman, N., & Dotan, D. (2023). Reading numbers is hard, and the difficulty is a syntactic one: A descriptive analysis of number-reading patterns in readers with and without dysnumeria. *PsyArXiv Preprints*. https://doi.org/10.31234/osf.io/87dzw

Hill, F., Mammarella, I. C., Devine, A., Caviola, S., Passolunghi, M. C., & Szűcs, D. (2016). Maths anxiety in primary and secondary school students: Gender differences, developmental changes and anxiety specificity. *Learning and Individual Differences*, *48*, 45–53. https://doi.org/10.1016/j.lindif.2016.02.006

Siregar, N. C., Rosli, R., & Nite, S. (2023). Students' interest in Science, Technology, Engineering, and Mathematics (STEM) based on parental education and gender factors. *International Electronic Journal of Mathematics Education*, *18*(2), em0736. https://doi.org/10.29333/iejme/13060
